# Predictors of utilization of facility-based ante-natal care and delivery services in a Nigerian Rural Community

**DOI:** 10.4314/ahs.v22i1.18

**Published:** 2022-03

**Authors:** Aniekan Etokidem, Iwasam Agbor, Anastasia Isika, Boniface Ago, Nkese Mkpanam

**Affiliations:** 1 Department of Community Medicine, University of Calabar, Calabar, Nigeria; 2 Department of Obstetrics and Gynaecology, University of Calabar, Calabar, Nigeria

**Keywords:** Maternal, Perinatal, Mortality, Child healthcare, Antenatal care, Delivery

## Abstract

**Background:**

With maternal mortality ratio of 2,000/100,000 live births and perinatal mortality rate of 40/1,000 total births, Cross River State is one of the states with the highest maternal and perinatal deaths in Nigeria. One of the causes of these poor health indices is low utilization of facility-based maternal and child healthcare services during pregnancy and childbirth. The objective of this study was to assess the predictors of utilization of antenatal care and delivery services in Akpabuyo, a rural community in Cross River State of Nigeria.

**Method:**

This was an analytical cross-sectional survey. Data were collected from 370 pregnant women between June and July, 2013 and analyzed using SPSS version 25.

**Results:**

Binary logistic regression showed that compared with women with tertiary education, women with non-formal education were less likely to attend antenatal clinic (AOR=0.510, 95% CI=0.219–1.188) although the difference was not statistically significant. Also, compared with farmers, full-time housewives were less likely to deliver in a health facility (AOR=0.650, 95% CI=0.305–1.389) while civil servants were nearly five times more likely to deliver in the health facility (AOR=4.750, 95%CI=1.616–13.962).

**Conclusion:**

The predictors of antenatal care and facility delivery services utilization identified by the study were educational status and occupation. This raises the need for policies and programmes to ensure girl child education and the economic empowerment of women.

## Introduction

Every day, approximately 810 maternal and 7, 000 neonatal deaths take place globally.^1^,^2^ Antenatal care (ANC) during pregnancy and attendance by a skilled healthcare provider during delivery can prevent these maternal and neonatal deaths as well as other adverse outcomes.^3^,^4^ Antenatal care is the care provided by skilled healthcare professionals to pregnant women in order to ensure the best health conditions for both mother and the newborn.^3^,^4^ A number of ANC models have been tried over time. The traditional model involved up to 7 to 16 visits to the healthcare provider and was a challenge in resource-constrained settings.^3^,^4^ The 2016 WHO ANC Model recommends a minimum of eight contacts, with the first contact scheduled to take place in the first trimester (up to 12 weeks of gestation), two contacts scheduled in the second trimester (at 20 and 26 weeks of gestation) and five contacts scheduled in the third trimester (at 30, 34, 36, 38 and 40 weeks).^4^ Skilled attendance at delivery is a benchmark indicator to measure progress towards reducing maternal mortality and morbidity. 5 Sustainable Development Goal 3 Target 3.1 is to reduce the global maternal mortality ratio to less than 70 per 100,000 live births by 2030 while Target 3.2 is to end preventable deaths of newborns and to reduce neonatal mortality to at least as low as 12 per 1,000 live births. 6

Nigeria's maternal mortality ratio of 576/100,000 live births is one of the highest in the world.^7^ Nigeria's perinatal and neonatal mortality rates of 49/1,000 total births and 39/1,000 live births respectively, are also unacceptably high.^7^ These poor maternal and child health indicators may be due to low utilization of facility-based services. For instance, only 67% of pregnant women in Nigeria received antenatal care compared to 96% in Ghana. 7, 8 Similarly, only 39 % of women delivered in health facilities in Nigeria compared to 95% in Botswana. 9

With a maternal mortality ratio of 2,000/100,000 live births and perinatal mortality rate of 40/1,000 total births, Cross River State is one of the states with the highest maternal and perinatal deaths in Nigeria.^10^

One of the causes of the high maternal, perinatal and neonatal morbidity and mortality experienced in Nigeria is low utilization of health facility-based maternal and child healthcare services during pregnancy, especially, antenatal care and assistance by a skilled birth attendant during delivery.^7^ During ANC, pregnant women are provided with relevant health education and undergo screening for risk factors for diseases and testing and treatment for existing health conditions. 2 Other ANC services include intermittent preventive treatment of malaria using sulfadoxine/pyrimethamine and screening for and treatment of anaemia. This averts the risk of adverse maternal outcome in the event of postpartum haemorrhage during delivery. There are also benefits to the fetus and newborn as it averts intra-uterine growth restriction, fetal death, perinatal and neonatal mortality as well as ensure optimal child growth. Skilled birth attendance during and immediately after delivery also provides opportunity for addressing conditions that may adversely affect the outcome of the delivery for both the mother and the baby.^2^,^11^

The Reproductive, Maternal, Neonatal, Child and Adolescent Health goal of Nigeria's Integrated Maternal, Newborn and Child Health Strategy and the National Strategic Health Development Plan is to ‘reduce maternal, neonatal, child and adolescent morbidity and mortality in Nigeria, and promote universal access to comprehensive sexual and reproductive health services for adolescents and adults throughout their life cycle.’ The targets are to reduce maternal mortality ratio to 288/100,000 live births, increase percentage of pregnant women attending eight ANC visits to 80%, increase skilled attendance at delivery to 57% and reduce neonatal mortality rate to 18/1,000 live births. 12,13 The Cross River State Strategic Health Development Plan 2010–2015 documented that although 68% of pregnant women received ANC provided by skilled healthcare providers, only 39% delivered in the health facility. 10 This calls for urgent action from individuals, policy makers, programmers and the government so that Cross River State and Nigeria can contribute meaningfully towards the attainment of SDG 3 targets 3.1 and 3.2. The objective of this study was to assess the predictors of utilization of antenatal care and delivery services in Akpabuyo, a rural community in Cross River State of Nigeria.

## Methods

### Study setting

This study took place in Akpabuyo, a rural Local Government Area (LGA) in Cross River State of Nigeria. This LGA shares boundaries with Akampa LGA, the Republic of Cameroon, Bakassi LGA, and Calabar South LGA and Calabar Municipality to the North, East, South and West respectively.

There are 10 political wards in the LGA. Akpabuyo is a local government area comprising several agrarian communities. It has a population of 360,000.^14^ The major ethnic groups are the Efiks, Quas and Efuts. Majority of the people are farmers while others are engaged in trading, fishing and civil service. There is a preponderance of traditional birth attendants and church-based maternity services in all communities in the LGA. The LGA is served by one Secondary healthcare facility, a General Hospital and 74 Primary Healthcare facilities. 15

### Study design

This was an analytical cross-sectional study.

### Study population

The study population consisted of pregnant women in Akpabuyo LGA.

### Inclusion criteria

Pregnant women who were fulltime residents in Akpabuyo and who gave consent to participate in the study.

### Exclusion criteria

Pregnant women who were visiting their relatives and therefore, not fulltime residents of the LGA. Fulltime resident pregnant women who refused to give consent were also excluded.

### Sample size determination

Using Cochran's formula and a prevalence of 68% 10 the sample size was calculated as 334 which, after making provision for 10% non-response rate, was approximated to 370.

### Sampling methodology

Simple random sampling was used to select eight out of ten wards in the LGA. Eight wards were randomly selected because the sample size could be obtained from this number of wards. The list of the ten wards in the LGA, numbered from one to ten, constituted the sampling frame. Simple random sampling of 8 out of 10 wards was done using a table of random numbers. The team then went to villages within each ward. In each village, a starting point was selected, usually, the palace of the village head. The team then selected a random starting point by spinning a bottle. The team started with the first household in the direction that the bottle pointed towards and moved from one household to another. In every household with pregnant women, all eligible ones who gave consent were interviewed. At the end of the direction, the team returned to the central location and repeated the process. If the bottle faced the same direction as before, the process was repeated until it faced a different direction. This was done from one village to another until the sample size for each ward was obtained.

### Study instrument and data collection

A semi-structured interviewer-administered questionnaire was used to collect data from the respondents. The study variables included sociodemographic information such as age, marital status, occupation, religion, ethnic group, educational status and family structure (monogamous or polygamous). There were questions on pregnancy-related and service utilization variables such as number of previous pregnancies, ANC attendance during the last pregnancy, place of delivery in the last pregnancy, gestational age in current pregnancy, gestational age at time of commencement of ANC in current pregnancy, intended place of delivery in the current pregnancy and reasons for non-utilization of facility-based ANC and delivery services, amongst other variables.

Forty questionnaires were pre-tested in Ikot Effiong Otop in Odukpani LGA. The pre-testing was done using face-to-face interview. Corrections were effected in the instrument after pre-testing. For the main data collection, face-to-face interviews were conducted by five trained and experienced research assistants who were supervised by a trained data collection supervisor. The authors also provided supportive supervision during data collection.

Data collection took place between June and July, 2013. Data collected from the field arrived the research office at the University of Calabar on daily basis. The data quality control officer cross-checked each instrument for completeness. Where there were errors and or clarifications to be made, the data collector was contacted on the phone.

### Data preparation and analysis

Each questionnaire was given a unique identification number. The data were entered into the Statistical Package for Social Sciences, (SPSS) version 25 software and screened and cleaned. There was case count and variable count. Each variable was then assigned a unique identifier. Existence of undesirable variable types was checked, for instance, string variables that should have been numeric (and vice versa) were identified and corrected. User missing values were specified. The data were then analyzed and presented as tables, beginning with frequency tables to give a general view of the variables. Relationship between categorical variables was assessed using chi square test. The level of significance was set a p-value of <0.05. Binary logistic regression was carried out to identify the predictors of ANC attendance in the current pregnancy. After performing bivariate analysis, variables with p values <0.05 namely, occupation, educational status and family structure (whether monogamous or polygamous) were fitted into the logistic regression model. The other variables, which were not statistically significant and therefore not fitted into the model were age group, religion and marital status. Odds ratios (both crude and adjusted) with their corresponding 95% Confidence Intervals were calculated.

Similarly, binary logistic regression was carried out to identify the predictors of intention to utilize facility-based delivery services in the current pregnancy. After performing bivariate analysis, variables with p values <0.05 (occupation and educational status) were fitted into the logistic regression model. The other variables, which were not statistically significant and therefore not fitted into the model were age group, religion, marital status and family structure (whether monogamous or polygamous). Odds ratios (both crude and adjusted) with their corresponding 95% Confidence Intervals were calculated.

### Ethical clearance

Ethical clearance for this study was given by the Cross River State Health Research Ethics Committee while verbal informed consent was obtained from the participants. The study instruments did not carry any identifiers like names and addresses of participants. Hard copies of data were securely locked up in a cupboard and only authorized researchers had access to the key. Electronic data were stored in pass-worded computer systems and only authorized persons had access to the password.

## Results

### Socio-demographic characteristics

As shown in table 1, the mean age of respondents was 24.4+ 6.2 years. Fifty percent of the respondents were in the 15–24 age group. The least, 14 (3.8%) respondents belonged to the < 14 years age group. Majority of respondents, 363 (98.1%) were Christians while 7 (1.9%) belonged to ‘other’ religious groups.

**Table 1:** Socio-demographic characteristics

Variable name	Frequency (n=370)	Percent
**AGE (in years)**		
**Mean=24.4+ 6.2**		
<14	14	3.8
15–24	185	50.0
25–34	148	40.0
35–44	23	6.2
**RELIGION**		
Christianity	363	98.1
Others	7	1.9
**OCCUPATION**		
Farming	58	15.7
Trading	164	44.3
Fulltime Housewife	65	17.6
Civil Servant	23	6.2
Others.	60	16.2
**EDUCATION**		
Non-formal	29	7.8
Primary	189	51.1
Secondary	129	34.9
Tertiary	23	6.2
**MARITAL STATUS**		
Single	78	21.1
Married	210	56.8
Separated	15	4.0
Co-habiting	67	18.1
**FAMILY STRUCTURE**		
Monogamous	316	85.4
Polygamous	54	14.6

The main occupation of t h e respondents was trading, 164 (44.3%) while only 23 (6.2%) were civil servants. Regarding educational status, 189 (51.1%) respondents had obtained Primary School education while 23 (6.2%) had obtained tertiary education. Two hundred and ten (56.8%) respondents were married while 15 (4.1%) were separated. Majority of respondents, 316 (85.4%) were into monogamous relationships.

### Ante-natal care and delivery services utilization

Majority of respondents, 305 (82.4%) had been pregnant at least once prior to the current pregnancy of which 284 (93.1%) had been pregnant between one and four times while 21 (6.9%) had been pregnant at least five times before. Regarding ANC attendance, 186 (61%) respondents had received facility-based ANC services in their last pregnancy while 273 (73.8%) were receiving the services in the current pregnancy and out of this, 107 (39.2%) commenced ANC visit within the first trimester. Regarding number of ANC visits, 164 (60.1%) respondents had between one and three ANC visits and 109 (39.9 %) had four or more visits as at the time of the study. Regarding delivery services, one hundred and twenty-nine (42.3%) respondents had their deliveries in the health facility in their last pregnancy. One hundred and seventy-three (46.8%) respondents had the intention to deliver their babies in the health facility in the current pregnancy. (Table 2)

**Table 2:** Ante-natal care and delivery services utilization

Variable	Frequency	Percent
**Pregnant before?**		
Yes	305	82.4%
No	65	17.6%
**Number of previous pregnancies** (n=305)		
1–4	284	93.1%
5 and above	21	6.9%
**ANC attendance**		
Previous pregnancy	186	61%
Present pregnancy	273	73.8%
**Number of times of ANC attendance in** **present pregnancy**		
< 4 times	164	60.1%
> 4 times	109	39.9%
**Timing of commencement of ANC in** **present pregnancy**		
First trimester	107	39.2%
Second trimester	153	56.0%
Third trimester	13	4.8%
**Place of delivery**		
In previous pregnancy		
Health facility	129	42.3%
Outside the health facility	176	57.7%
**Intended place of delivery in present** **pregnancy**		
Health facility	173	46.8%
Outside the health facility	197	53.2%

Reasons for not utilizing health facility-based ANC and delivery services (Table 3 and figure I)

**Table 3:** Reasons for non-utilization of facility-based services

Reasons for non-utilization of facility-based ANC services in present pregnancy	Frequency (n=97)	Percent
I was being attended to by traditional birth attendant	32	33%
Attending church midwifery home /Other midwifery homes	40	41.2%
Visited at home by nurse/other health care provider	16	16.5%
Visited at home by village health worker (VHW)	2	2.1%
Health facility is too far	27	27.8%
I had transportation problems	15	15.5%
It is against my cultural beliefs	8	8.3%
It is against my religious belief	29	29.9%
There was no money to pay	14	14.4%
Poor attitude of health care providers	80	82.4%
Delay in the health facility	46	47.4%
Lack of consent from my husband/other relative	18	18.6%
Lack of consent from my relative(s)	14	14.4%
Other reason(s)	30	31%

**Figure I:**
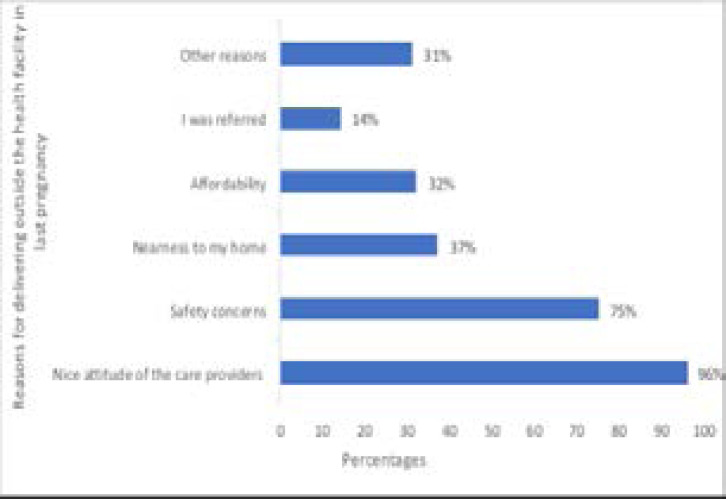
Reasons for delivering outside the health facility in the last pregnancy

Some of the reasons given for not utilizing facility-based ANC services were: poor attitude of healthcare providers, 80 (82.4%); delay in the facility, 46(47%) and attending church midwifery home or other midwifery homes, 40 (41.2%). Some reasons for preference of delivery outside the health facility included ‘nice attitude of care providers’ (that is traditional/ other birth attendants), 169 (96%); ‘safety concerns’, 132(75%) and ‘nearness to my home’ 65(37%).

### Predictors of utilization of facility-based ANC services in the current pregnancy

As shown in table 5, educational level was a positive predictor of utilization of ANC services. Compared with women with tertiary education, women with non-formal education were less likely to attend antenatal clinic (AOR=0.510, 95% CI=0.219–1.188). Family structure was also a positive predictor of ANC utilization. Compared with women in monogamous relationships, women in polygamous relationships were more likely to attend antenatal clinic, although the difference was not statistically significant. (AOR=2.307, 95% CI=0.948–5.612). Occupation was also a positive predictor of utilization of ANC services and was statistically significant. Compared with farmers, civil servants were nearly four times more likely to attend antenatal clinic (AOR=3.735, 95% CI=1.234–11.301).

**Table 5:** Binary logistic regression of ANC attendance in the current pregnancy as dependent variable with statistically significant variables from bivariate analysis

Variable	Crude OR	95% CI	Adjusted OR	95% CI
**Occupation** a				
Farming	Ref			
Trading	3.686	2.537–5.355	1.310	0.634–2.706
Fulltime Housewife	4.909	2.567–9.388	2.696	1.129–6.438
Civil Servant	4.750	1.616–13.962	3.735	1.234–11.301
Others	1.609	0.956–2.707	0.719	0.321–1.613
**Educational status** b				
Non-formal	0.611	0.289–1.294	0.510	0.219–1.188
Primary	2.436	1.780–3.335	1.879	1.029–3.429
Secondary	5.450	3.383–8.779	3.483	1.582–7.668
Tertiary	Ref			
**Family structure** c				
Monogamous	Ref			
Polygamous	6.714	3.035–14.854	2.307	0.948–5.612

a adjusted for educational status and family structure

b adjusted for occupation and family structure

c adjusted for occupation and educational status

OR=Odds Ratio, CI=Confidence Interval, Ref=Reference category

### Predictors of intention to utilize facility-based delivery services in the current pregnancy

As shown in table 7, educational level was a positive predictor of intention to deliver in a health facility. Compared with women with tertiary education, women with non-formal education were less likely to intend to deliver in the health facility in the current pregnancy (AOR=0.297, 95% CI=0.115–0.765). Occupation was a statistically significant positive predictor of intention to deliver in a health facility. Compared with farmers, fulltime housewives were less likely to deliver in a health facility (AOR=0.650, 95% CI=0.305–1.389) while civil servants were nearly five times more likely to deliver in the health facility (AOR=4.750, 95%CI=1.616–13.962)

**Table 7:** Binary logistic regression of intended place of delivery in the current pregnancy as dependent variable with statistically significant variables from bivariate analysis

Variable	Crude OR	95% CI	Adjusted OR	95% CI
**Occupation** a				
Farming	Ref			
Trading	1.000	0.736–1.358	1.065	0.547–2.072
Fulltime Housewife	0.548	0.329–0.910	0.650	0.305–1.389
Civil Servant	4.750	1.616–13.962	4.750	1.616–13.962
Others	0.714	0.428–1.193	0.866	0.406–1.847
**Educational status** b				
Non-formal	0.261	.106–0.641	0.297	0.115–0.765
Primary	0.835	0.627–1.112	0.907	0.510–1.612
Secondary	0.925	0.655–1.307	0.970	0.488–1.927
Tertiary	Ref			

a adjusted for educational status

b adjusted for occupation

## Discussion

This study assessed the predictors of utilization of antenatal care and delivery services in Akpabuyo, a rural community in Cross River State of Nigeria. Educational status was found to be a positive predictor of utilization of ANC services. A study by Umar et al in Yobe State in Northern Nigeria found a similar relationship between education and ANC attendance.^16^ A similar study in Bangladesh also found that the higher educated adult women were more likely to have received antenatal care four or more times than women with lower education.^17^ Onasoga et al also found a positive relationship between education and ANC attendance in Osun State, South-West Nigeria just as was found by Howlader et al in Dhaka city of Bangladesh.^18^,^19^ Educational level was a positive predictor of intention to deliver in a health facility. This finding is similar to that of Lera et al which found that the strongest predictor of intention to use institutional delivery among pregnant women in Southern Ethiopia was prior information about delivery places.^20^

One hundred and seventy-three (46.8%) respondents intended to deliver their babies in the health facility, a proportion which was less than the 74.3% recorded in a study in North-West Ethiopia.^21^ The low proportion in this study may be because the Ethiopian study took place in an urban setting while this study took place in a rural area. Urban dwellers are more likely to be educated than rural dwellers and, therefore, may have a better health-seeking behavior. Education increases both an individual's health literacy and health-seeking behavior.^22^

The study found that ANC attendance in the previous pregnancy was 61% while that of the current pregnancy was 73.8%. Both proportions were below the recommended 90% ANC coverage which is necessary for meaningful reduction in maternal and newborn mortality.^23^ Both proportions were higher than the 55.4% documented in a study in Kenya.^24^ The study in Kenya was carried out in a health facility while the present study was community-based. The proportion of ANC attendance in the previous pregnancy was lower than the 84.6% recorded in a previous study in Sagamu, South Western Nigeria. 25 This may be because generally, South West Nigeria has the best maternal and child healthcare indices in Nigeria.^7^ Only 39.2% of respondents commenced their ANC in the first trimester, a proportion which was, however, higher than the 22% documented in a study in South Africa 26 and was similar to the findings of an earlier study in Cross River State. 27 The South African study was facility-based while this study was community-based. Most of the respondents, 56%, commenced their ANC in the second trimester, a finding which is similar to that of the Sagamu study. 25 The proportion of respondents who commenced their ANC in the third trimester, 4.8% was lower than the 11% reported in the South African study. 26 That majority of respondents failed to commence their ANC in the first trimester may mean that they do not have sufficient health education regarding the importance of this key element of maternal and child healthcare. This may also be an indication that the study population may have low health-literacy and therefore poor health-seeking behavior. This portrays a need for the Primary Healthcare Department to identify and train community members as Village Health Workers (VHWs). The VHWs would then be assigned to carry out health education and other behavior change communication activities among pregnant women within their communities. This would lead to an improvement in their health literacy and health-seeking behavior. Additionally, the healthcare providers should spend more time in the communities, embarking on health promotion activities.

The low utilization of ANC and facility-based delivery services poses a threat to the state's attainment of the SDG 3 targets 3.1 and 3.2. It indicates a need for policy and programmatic intervention. Antenatal care and delivery services should be provided free, at least at the Primary Healthcare level. There is need to also introduce incentives for women who attend ANC and also deliver in the health facility. These could be monetary and non-monetary incentives such as free long-lasting insecticidal nets (LLINs), pampers for the babies, antimalarials and haematinics. Skilled birth attendants should also be motivated for attending a given number of deliveries.

Traditional birth attendants supervised 45.7% of the deliveries, a proportion which was lower than the 60% documented in Ogun State of Nigeria.^28^ TBAs supervised more deliveries than skilled healthcare providers in this study area.

The low proportion of respondents who delivered in a health facility in the previous pregnancy and of those who intended to do so in the current pregnancy could be due partly to their dissatisfaction with the attitude of healthcare providers. This assertion is corroborated by the finding that 82.4% of those who delivered outside the health facility indicated that they did so because of the poor attitude of the healthcare providers. The negative impact of poor health care provider attitude on utilization of maternal and child healthcare services have been documented by several other studies. 29–32 The State Ministry of Health and the LGA PHC Department should carry out attitudinal re-orientation training for the healthcare providers. There may be need for the carrot and stick approach where healthcare providers with welcoming attitude to their clients are rewarded while those whose attitude cause client dissatisfaction are punished.

More respondents who were co-habiting utilized facility ANC services than those who were married. The proportion who also intended to deliver in the health facility was comparable to that of married women and higher than those of single and separated respondents. These high proportions may be because of the cultural practice in this part of the country whereby, if a woman in this type of relationship dies during pregnancy, the man would face severe sanctions from the family of the woman. In some instances, the male partners have been forced to ‘marry’ the corpses of the women who were co-habiting with them. The fear of such sanctions may cause the man to encourage the woman co-habiting with him to utilize health facility ANC and delivery services.

## Conclusion

The study identified educational status and occupation as predictors of ANC attendance and intention to utilize facility-based child delivery services. This raises the need for policies and programmes to ensure girl child education and economic empowerment of women as these would improve their health literacy, health-seeking behavior and ability to afford the cost of healthcare.

## Strengths of the study

The strength of this study was that it was community-based as different from a facility-based study. The setting of the study therefore removed the fear that respondents could have of a possibility of healthcare providers being aware of the answers they provided. Such fear could lead to biased responses.

## Limitations of the study

This study could have benefited from a qualitative methodology. This could not be done due to resource limitation. The study was done in only one out of 18 LGAs in the state. This is a limitation regarding the generalizability of its findings to the entire state.

## Figures and Tables

**Table 4:** Bivariate Analysis: Association between socio-demographic variables and utilization of ANC services in the current pregnancy

Variable	ANC Attendance		Chi square	P-value
	Yes	No		
**AGE (in years)**				
<14	9 (64.3%)	5 (35.7%)		
15–24	133 (71.9%)	52(28.1%)	5.103	>0.05
25–34	117 (79.1%)	31 (20.9%)		
35–44	14 (60.9%)	9(39.1%)		
**RELIGION**				
Christianity	270 (74.4%)	93 (25.6%)	3.528	>0.05
Others	3 (42.9%)	4 (57.1%)		
**OCCUPATION**				
Farming	34 (58.6%)	24 (41.1%)		
Trading	129 (78.7%)	35 (21.3%)		
Fulltime Housewife	54(83.1%)	11 (16.9%)	17.291	<0.05*
Civil Servant	19 (82.6%)	4(17.4%)		
Others	37 (61.7%)	23 (38.3%)		
**EDUCATIONAL** **STATUS**				
Non-formal	11 (37.9%)	18 (62.1%)		
Primary	134 (70.9%)	55 (29.1%)	28.663	<0.001***
Secondary	109 (84.5%)	20 (15.5%)		
Tertiary	19 (82.6%)	4 (17.4%)		
**MARITAL STATUS**				
Single	53 (67.9%)	25 (32.1%)		
Married	158 (75.2%)	52 (24.8%)	64.54	>0.05
Separated	8 (53.3%)	7 (46.7%)		
Co-habiting	54 (80.6%)	13 (19.4%)		
**FAMILY STRUCTURE**				
Monogamous	226 (71.5%)	90 (28.5%)	5.741	<0.05*
Polygamous	47 (87.0%)	7 (13.0%)		

Not Significant (P > 0.05)

* Significant (P < 0.05)

*** Highly Significant (P < 0.001)

**Table 6:** Bivariate Analysis: Association between socio-demographic variables and intended place of delivery in the current pregnancy

Variable	Intended place of delivery		Chi square	P-value
	Health facility	Other places		
**AGE (in years)**				
<14	6 (42.9%)	8(57.1%)		
15–24	89 (48.1%)	96(51.9%)	0.360	>0.05
25–34	68 (45.9%)	80(54.1%)		
35–44	10(43.5%)	13(56.5%)		
**RELIGION**				
Christianity	171 (47.1%)	192 (52.9%)	0.948	>0.05
Others	2(28.6%)	5(71.4%)		
**OCCUPATION**				
Farming	24 (41.4%)	34 (58.6%)		
Trading	82 (50.0%)	82(50.0%)		
Fulltime Housewife	23(35.4%)	42 (64.6%)	17.243	<0.05*
Civil Servant	19 (82.6%)	4(17.4%)		
Others	25 (41.7%)	35 (58.3%)		
**EDUCATIONAL** **STATUS**				
Non-formal	6 (20.7%)	23 (79.3%)		
Primary	86 (45.5%)	103 (54.5%)	19.998	<0.001***
Secondary	62 (48.1%)	67 (51.9%)		
Tertiary	19 (82.6%)	4 (17.4%)		
**MARITAL STATUS**				
Single	32 (41.0%)	46 (59.0%)		
Married	105 (50.0%)	105 (50.0%)	4.375	>0.05
Separated	4 (26.7%)	11 (73.3%)		
Co-habiting	32(47.8%)	35 (52.2%)		
**FAMILY STRUCTURE**				
Monogamous	153 (48.4%)	163 (51.6%)	2.399	>0.05
Polygamous	20 (37.0%)	34 (63.0%)		

Not Significant (P > 0.05)

* Significant (P < 0.05)

*** Highly Significant (P < 0.001)
